# Safety and Complications of Medical Thoracoscopy

**DOI:** 10.1155/2016/3794791

**Published:** 2016-06-19

**Authors:** Shimaa Nour Moursi Ahmed, Hideo Saka, Hamdy Ali Mohammadien, Ola Alkady, Masahide Oki, Yoshimasa Tanikawa, Rie Tsuboi, Masahiro Aoyama, Keiji Sugiyama

**Affiliations:** ^1^Department of Respiratory Medicine, National Hospital Organization Nagoya Medical Center, Nagoya, Aichi 460-0001, Japan; ^2^Department of Respiratory Medicine, Sohag University Hospital, Nasr City Street, Sohag 82524, Egypt; ^3^Department of Respiratory Medicine and Clinical Immunology, Toyota Kosei Hospital, 500-1 Ibohara Josuicho, Toyota-Shi 470-0396, Japan

## Abstract

*Objectives.* To highlight the possible complications of medical thoracoscopy (MT) and how to avoid them.* Methods.* A retrospective and prospective analysis of 127 patients undergoing MT in Nagoya Medical Center (NMC) and Toyota Kosei Hospital. The data about complications was obtained from the patients, notes on the computer system, and radiographs.* Results.* The median age was 71.0 (range, 33.0–92.0) years and 101 (79.5%) were males. The median time with chest drain after procedure was 7.0 (range, 0.0–47.0) days and cases with talc poudrage were 30 (23.6%). Malignant histology was reported in 69 (54.3%), including primary lung cancer in 35 (27.5), mesothelioma in 18 (14.2), and metastasis in 16 (12.6). 58 (45.7%) revealed benign pleural diseases and TB was diagnosed in 15 (11.8%). 21 (16.5%) patients suffered from complications including lung laceration in 3 (2.4%), fever in 5 (3.9%) (due to hospital acquired infection (HAI) in 2, talc poudrage in 2, and malignancy in 1), HAI in 2 (1.6%), prolonged air-leak in 14 (11.0%), and subcutaneous emphysema in 1 (0.8%).* Conclusions.* MT is generally a safe procedure. Lung laceration is the most serious complication and should be managed well. HAI is of low risk and can be controlled by medical treatment.

## 1. Introduction

MT is used increasingly by chest physicians and has become, after bronchoscopy, the second most important endoscopic technique in respiratory medicine [1]. It is considered to be one of the main areas of interventional pulmonology and an important part of a specialist pleural disease service [2, 3].

Thoracoscopy is the oldest invasive interventional technique in the recent history of respiratory medicine. It was initially performed in 1910 by an internist from Sweden, Hans-Christian Jacobaeus. Jacobaeus was the first to use the term thoracoscopy that he described as “replacing fluid with air” in order to examine the pleural surfaces of two patients with tuberculous pleurisy. Jacobaeus later developed a therapeutic application for thoracoscopy by using thermocautery to lyse adhesions and create a pneumothorax to treat tuberculosis (Jacobaeus operation) [4].

At the end of the 1990s, semirigid (or flex-rigid) thoracoscope was successfully introduced as a new instrument for thoracoscopy [5]. Pulmonologists who used to work with a flexible bronchoscope found it more familiar. It allows easy lateral vision or even retro visualization of the point of entry, but its main limitations are small biopsies taken through the small working channel and the difficulty to lyse the adhesions [6].

The application of MT is mainly for diagnosing pleural effusion and for performing talc poudrage pleurodesis in malignant pleural effusion and recurrent spontaneous pneumothorax [7]. For the diagnosis of malignant pleural disease, MT has consistently been demonstrated to be superior to fluid cytology and “blind” closed needle biopsy [8–10] with a sensitivity of up to 95% [11]. A recent study comparing MT with CT-guided Abrams needle biopsy demonstrated no significant difference with the two techniques, although the advantage of thoracoscopy remains that pleurodesis can be performed after pleural fluid removal during the procedure [12].

It also plays a crucial role in staging non-small-cell lung cancer and guiding treatment and prognosis, as the documentation of pleural metastasis renders the patient inoperable (stage M1a) [13]. It can be useful as well to provide large biopsies required for the application of molecular techniques, such as the use of molecular markers, for example, epidermal growth factor receptor; these markers participate in the modern staging of malignant diseases and provide possibilities for potential therapies [14]. Other nonroutine and more complex applications of MT are in the treatment of empyema, in lung biopsy with forceps, and in cervical sympathectomy; these procedures are considered advanced, need more experience, and should definitely be performed by experts and highly trained thoracoscopists [15].

MT in the hands of experienced physicians is safe with mortality of 0.35% (95% confidence interval (CI) 0.19–0.54%) and likely to be less if diagnostic procedures alone are performed [16]. This compares favorably with mortality from transbronchial biopsy (0.22–0.6%) [17] and mediastinoscopy (0.17%) [18]. Pain is frequently reported after the procedure and may be more common when using talc poudrage for pneumothorax when compared with malignant effusion [19]. There has been previous concern with respect to the development of acute respiratory distress syndrome (ARDS) following talc poudrage, [20] which was likely to be from the grade of talc utilized [21]. Major complications (empyema, hemorrhage, port site tumor growth, bronchopleural fistula and/or persistent air leak, postoperative pneumothorax, and pneumonia) occur in 1.8% (95% CI 1.4–2.2%) and minor complications (subcutaneous emphysema, minor hemorrhage, operative skin site infection, fever, and atrial fibrillation) occur in 7.3% (95% CI 6.3–8.4%) [16].

The aim of this study is to evaluate the safety of MT and the possible complications that can occur during and after the procedure.

## 2. Materials and Methods

### 2.1. Patients

We performed a retrospective and prospective analysis of 127 patients undergoing MT for diagnostic and therapeutic purposes in NMC during the period of 2007 to 2015 and Toyota Kosei Hospital during the period of 2011 to 2015. The retrospective analysis included patients during the period of 2007 to 2013 in NMC and during the period of 2011 to 2013 in Toyota Kosei Hospital and prospective analysis included the patients during 2014 and 2015 for both.

During the period of our study, the total number of patients was 139 but we excluded 10 patients due to defective data required for analysis and the other 2 patients were excluded due to failure to complete MT. The patients' data was obtained from the hospital computerized system. The case notes were examined for any report of complications and radiographs were examined for outcome. The histology and relevant microbiology reports were obtained from the laboratory computerized system.

### 2.2. MT and Chest Tube Management

MT was performed by experienced physicians using The Olympus semirigid LTF-260 thoracoscopy. The patients were in the lateral position with local anesthesia and conscious sedation using midazolam and fentanyl in NMC and only local anesthesia in Toyota Kosei Hospital, with ECG and pulse oximetry monitoring throughout.

About 5 to 10 biopsies were taken from the parietal pleura depending on the macroscopic findings and 4 g of graded talc (Steritalc®, Novatech, La Ciotat, France) poudrage was used during pleurodesis. All patients had 16 F chest drains after the procedure through the same entry port as the thoracoscopy. Intercostal drains were connected to an underwater seal and negative suction (−10 to −20 cm/H_2_O) was used till the drained fluid becomes less than 150 mL/day.

### 2.3. Prethoracoscopic Investigations

All patients were subjected to pleural fluid analysis, chest X-ray, chest CT, chest ultrasonography (US), ECG, and routine liver and kidney functions.

### 2.4. Thoracoscopic Technique

*Premedication.* Midazolam 0.4 mg together with fentanyl 0.2 mg was administered to the patients just before the procedure through intravenous line and sometimes during the procedure if it lasted for long time.

Oxygen via nasal cannula was administered during the entire procedure to maintain SpO_2_ >90%.

After premedication, the combined scope and forceps were inserted, under local anesthesia, through a trocar with an outer diameter of 10 mm and without valve, in an intercostal space, with the patient resting on the healthy side. A sterile catheter was introduced through the trocar and the residual effusion was taken under sterile conditions. After the pleural cavity had been emptied, the lung and pleura were examined by way of the thoracoscope and any abnormality like adhesions, fibrin deposition, discoloration of the pleura, hyperemia, tuberculous nodules, formation of tumors, and so forth was observed.

Whenever feasible, biopsies were taken, placed immediately in formalin, and sent for microscopic examination. About 5 to 10 biopsies were taken from each patient from the parietal pleura depending on the macroscopic findings.

### 2.5. Statistical Analysis

Statistical analyses were processed by statistical software program (PASW Statistics 16; SPSS Inc., Chicago, IL, USA). Data were statistically described in terms of frequencies (number of cases), percentages, median, and range when appropriate.

## 3. Results

The total number of cases was 127 with median age of 71.0 (range, 33.0–92.0) years and 101 (79.5%) were males; as regards smoking, 36 (28.3%) cases were current smokers. The median duration of drainage by intercostal tube after MT was 7.0 (range, 0.0–47.0) days and cases of talc poudrage were 30 (23.6%). The details of patients' characteristics are shown in Table 1.

Benign pleural disease was diagnosed in 58 (45.7%), including tuberculosis in 15 (11.8%), empyema in 10 (7.9%), the nonspecific pleuritis in 27 (21.3%), hypoalbuminemia in 2 (1.6%), uremic pleuritis in 2 (1.6%), drug induced pleuritis in 1 (0.9 %), and ruptured bulla in 1 (0.9 % of total). The details are shown in Table 2.

Malignant histology was reported in 69 (54.3%) including primary lung cancer in 35 (27.5%), metastasis in 16 (12.6%), and mesothelioma in 18 (14.2%). The frequency of different histological types of lung cancer was adenocarcinoma in 29 (82.9%), squamous-cell carcinoma in 4 (11.4%), and small-cell carcinoma in 2 (5.7%). The most common source of metastasis was lymphoma in 4 (25%). The details are shown in Tables 2 and 3.

No mortality was related to the procedure of MT. No major bleeding from the biopsy sites or hemoptysis was observed. Complications occurred in 21 (16.5%) cases including lung lacerations in 3 (2.4%), fever in 5 (3.9%) (due to HAI in 2, talc poudrage in 2, and malignancy in 1), HAI in 2 (1.6%), prolonged air leak in 14 (11.0%), and subcutaneous emphysema in 1 (0.8%). The details are shown in Table 4.

MT failed in two cases: the first case was due to extensive adhesions which rendered introducing the MT and the second one was a case of left primary spontaneous haemopneumothorax due to continuous bleeding from the ruptured emphysematous bulla.

## 4. Discussion

**Figure 1:**
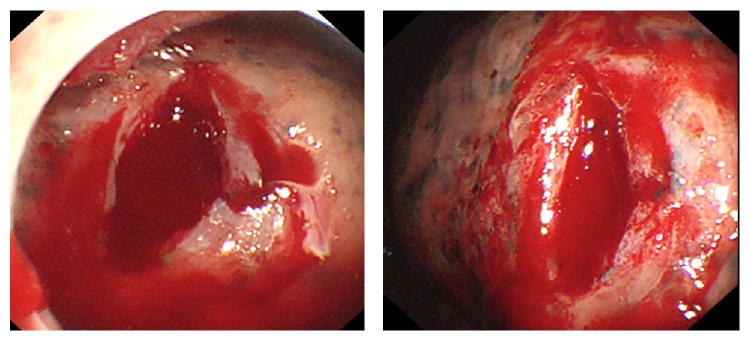
Lung laceration in one of the patients following introducing the trocar.

Although this study was done on a large number of patients (127), the rate of complications was not so severe 16.5% (*n* = 21). The most serious complication was lung laceration which occurred in 3 cases, all of them occurred during introducing the trocar and did not exceed 1.5 cm in length as illustrated in Figure 1. Two of them were managed during performing MT by calling the cardiothoracic surgeon and the application of polyglycolic acid (PGA) mesh and fibrin glue over the site of the laceration under local anesthesia without any complications during and after the procedure but the third case was neglected during the procedure and that resulted in air leak and subcutaneous emphysema after procedure which increased and indicated urgent surgical intervention to close the laceration.

To prevent the occurrence of lung laceration, some authors advocate the creation of pneumothorax a few hours or even the day before the thoracoscopy. However, direct introduction of a blunt trocar into the thoracic wall without prior induction of pneumothorax is safe and effective, especially if there is enough pleural fluid. The trocar should always be inserted perpendicular to the chest wall with a rotating motion. It is safer to locate the tip over the border of the inferior rib at the chosen port of entry, in order to prevent damage to the intercostal vessels and nerves. The introduction of the trocar can be troublesome especially in cases of pleural adhesions. When the physician is not sure about the presence of tight adhesions between the lung and the chest wall at the chosen site of entry, a digital dissection and direct exploration of the port of entry with the telescope may be helpful [22]. Chest US can be also very helpful to identify loculations in the pleural cavity and to locate the best entry site for thoracoscopy [23].

Actually, US was done before MT in the 3 cases complicated with lung lacerations, pneumothorax was created before introducing MT, and there were no extensive adhesions which may play a role in the occurrence of lung laceration, but the main cause was vigorous maneuver during introducing the trocar.

The second and important complication in this study was fever; it occurred in 5 cases due to different causes. Fever was grade 1 in all cases according to Common Terminology Criteria for Adverse Events (CTCAE) version 4.0. Two of them occurred after talc poudrage and lasted only for two days and improved with antipyretics. The third case developed fever due to malignancy; this patient was diagnosed as pleural metastasis from renal cell carcinoma and did not improve with antipyretics or antibiotics. Already the patient presented with fever before performing MT, and it lasted with him until the discharge with exclusion of other causes. The fourth and fifth cases developed HAI in the form of worsening of the already diagnosed empyema after improvement following MT which was done for drainage and adhesiolysis; the fourth patient was of old age (70 years old) with bad general condition and stayed in hospital for one month; he developed fever two days after the procedure and improved after one week with antibiotics. The fifth patient has the same situation like the fourth one (being 76 years old, bad general condition, and long hospital stay for 33 days). He was complicated with septicemia and developed fever three days after the procedure which lasted for 2 days and then the patient died. Prolonged hospital stay with the bad general condition may play important role in the development of HAI.

There were 11 cases of death but not related to MT; the main cause was malignancy and the presence of other comorbidities as hepatic and renal diseases.

The median time for drainage by intercostal tube after procedure was 7.0 (range, 0.0–47.0) days. In this study, long drainage time was due to prolonged air leak and incomplete lung reexpansion especially in neoplastic patients and patients diagnosed as empyema with other comorbidities and bad general condition. Cases complicated with prolonged air leak were 14 (11.0%) including 7 cases with malignancy, 6 cases with empyema, and one case with TB.

With respect to developing postprocedure infections, this represents an area that can potentially be modified by improved patient management immediately after the procedure including active chest physiotherapy and early mobilization to reduce basal atelectasis, all practices that can be learnt from our surgical colleagues [23, 24]. The use of a one-off dose of intravenous broad spectrum antibiotic at the time of the procedure may also lead to a reduction of infections following thoracoscopy, although this practice varies among physicians and was not routine practice during the study period. The rates in this study of empyema, pneumonia, and 1-month mortality compare favorably with other large thoracoscopy series [25, 26].

**Figure 2:**
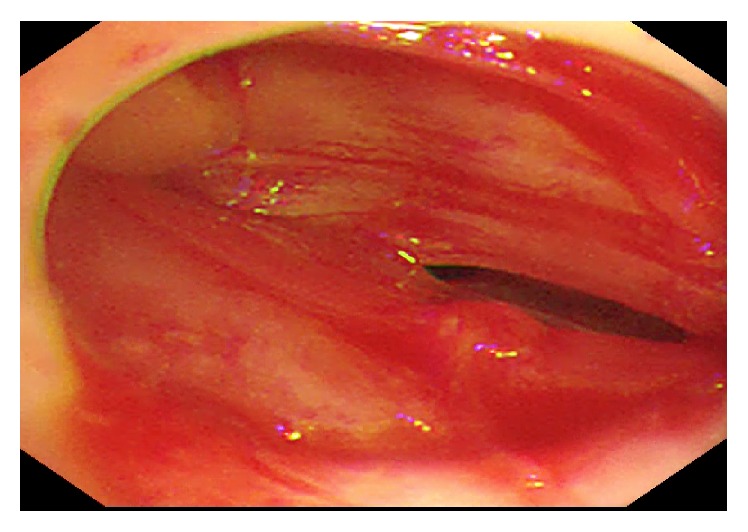
Obliteration of the pleural space due to extensive adhesions and pleural thickening.

MT failed in two cases: the first case was due to the presence of severe adhesions which rendered introducing MT so we gave up MT for the safety of the patient and thoracotomy was done. Chest US was used to determine the best site for introducing the trocar but the presence of extensive adhesions caused obliteration of the pleural cavity as illustrated in Figure 2. The second was a case of primary left haemopneumothorax due to spontaneous rupture of an emphysematous bulla, which was diagnosed before MT by chest CT. This indicated the presence of large amount of left pleural effusion with a rim of pneumothorax and a large apical emphysematous bulla, and thoracocentesis revealed hemorrhagic pleural effusion. The findings of bloody pleural effusion and normal pleura were observed during the procedure. Active bleeding was not clear but over 2,000 mL of hemorrhagic fluid was drained. A semiurgent video assisted thoracoscopic surgery (VATS) was indicated after the procedure, which was done on the next day for resection of the bulla.

## 5. Conclusions

In conclusion, medical thoracoscopy is generally a safe procedure. Lung laceration is the most serious complication and should be managed well. HAI is of low risk and can be controlled by medical treatment.

## Figures and Tables

**Table 1:** Characteristics of patients undergoing medical thoracoscopy.

Characteristics	The total number of patients 127
Age, median (range), year	71.0 (33.0–92.0)
Sex, no. (%)	
Male	101 (79.5)
Female	26 (20.5)
Smoking status, number (%)	
Nonsmoker	44 (34.6)
Smoker	36 (28.3)
Ex-smoker	47 (37)
The affected side, number (%)	
Right	69 (54.3)
Left	55 (43.3)
Bilateral	3 (2.4)
Nature of pleural fluid analysis, number (%)	
Exudate	124 (97.6)
Transudate	3 (2.3)
Duration of ICT drainage, median (range), days	7.0 (0.0–47.0)
Talc poudrage, number (%)	30 (23.6)

ICT: intercostals tube.

NB: 10 of the cases of exudate were purulent and one was hemorrhagic fluid.

**Table 2:** Diagnostic outcome of medical thoracoscopy in 127 patients.

Malignant (*n* = 69; 54.3% of total)	Benign (*n* = 58; 45.7% of total)
Primary lung cancer **35 **(27.5% of total)	Nonspecific pleuritis **27** (21.3% of total)
Metastasis to pleura **16** (12.6% of total)	Tuberculosis **15 (**11.8% of total)
Mesothelioma **18 **(14.2% of total)	Empyema **10** (7.9% of total)
	Hypoalbuminemia **2** (1.6% of total)
	Uremic pleuritis** 2 **(1.6% of total)
	Drug induced pleuritis **1** (0.9% of total)
	Ruptured bulla **1** (0.9% of total)

NB: Cases of empyema were diagnosed before doing MT; they were not postprocedure complications.

**Table:** (a) Differentiation of the primary lung cancers (*n* = 35)

Type	Number	(%) of lung cancer
Adenocarcinoma	29	(82.9)
Squamous cell carcinoma	4	(11.4)
Small-cell lung cancer	2	(5.7)

**Table:** (b) Primary origin of the metastases (*n* = 16)

Type	Number	(%) of metastases
Lymphoma	4	(25)
Kidney	2	(12.5)
Prostate	2	(12.5)
Ovary	2	(12.5)
Pancreas	2	(12.5)
Pharynx	1	(6.3)
Colon	1	(6.3)
Bladder	1	(6.3)
Thymus	1	(6.3)

**Table 4:** Thoracoscopic complications.

Total number of patients 127, patients with complications 21 (16.5%)	
Type	*N* (%) of total

Lung laceration	3 (2.4)
Fever	5 (3.9)
HAI	2 (1.6)
Prolonged air leak	14 (11.0)
Subcutaneous emphysema	1 (0.8)
Bleeding	0
ARDS	0
Mortality due to procedure	0

HAI, hospital acquired infection; ARDS, acute respiratory distress syndrome.

## Abbreviations

MT: Medical thoracoscopy
HAI: Hospital acquired infection
ARDS: Acute respiratory distress syndrome
US: Ultrasonography.
